# Death of a Healthcare Member: A Systematic Guide for Hospital Systems

**DOI:** 10.7759/cureus.97936

**Published:** 2025-11-27

**Authors:** Joseph A Demirjian, Bethany Bruzzi, Melissa Zukowski, Jessica Bates, Lisa Stoneking

**Affiliations:** 1 Emergency Medicine, University of Arizona College of Medicine - Tucson, Tucson, USA; 2 Family Medicine, University of Arizona College of Medicine - Tucson, Tucson, USA; 3 Emergency Medicine/Pediatric Emergency Medicine, University of Arizona College of Medicine - Tucson, Tucson, USA

**Keywords:** death, grief and bereavement, guidelines, healthcare systems, health system development, hospital management, management guideline

## Abstract

The death of a healthcare team member often has a significant impact on co-workers, hospital systems, friends, and family. A large community can be affected by grief, shock, and stress when faced with the tragedy of death, and there is limited information about the resources and guidelines that should be initiated. In the face of inadequate guidelines, we present a flexible procedural framework designed to effectively manage the series of events after the death of a healthcare team member occurring within or outside the hospital premises. This framework was developed within a prominent level 1 trauma academic medical center situated within a comprehensive healthcare organization. Our proposed outline compiles a contact list and a flow diagram for systematic notification of staff who may be involved in the hospital system during such an event. We reviewed relevant editorials and publications that help inform our method. During this heightened time of stress and grief, we believe it is imperative to consider patient care coverage, privacy and risk mitigation, and the well-being of other team members. Circumstances surrounding the death of team members may vary, but a well-developed, prepared, and organized approach may help avoid miscommunication, reduce confusion, and provide needed services throughout the bereavement period.

## Introduction

We present a flexible procedural framework designed to effectively manage the series of events after the death of a healthcare team member occurring either within the hospital premises or outside of it. Unfortunately, those in healthcare are not immune to the tragedy of losing a colleague, and our goal is to make a very difficult time easier by creating these procedures. Recognizing a lack of standardized institutional guidance, we conducted an iterative process involving stakeholder feedback, review of organizational policies, and targeted literature review [1-3]. Our goal was to create a well-developed, prepared, and organized approach that may avoid miscommunication, reduce confusion, and help provide services throughout the bereavement period. This framework was developed within a prominent level 1 trauma academic medical center situated within a comprehensive healthcare organization.

## Materials and methods

This framework was developed at an urban academic medical center following the in-hospital death of a healthcare team member. Recognizing a lack of standardized institutional guidance, we conducted an iterative process involving stakeholder feedback, review of organizational policies, and targeted literature review [1-3].

**Figure 1:**
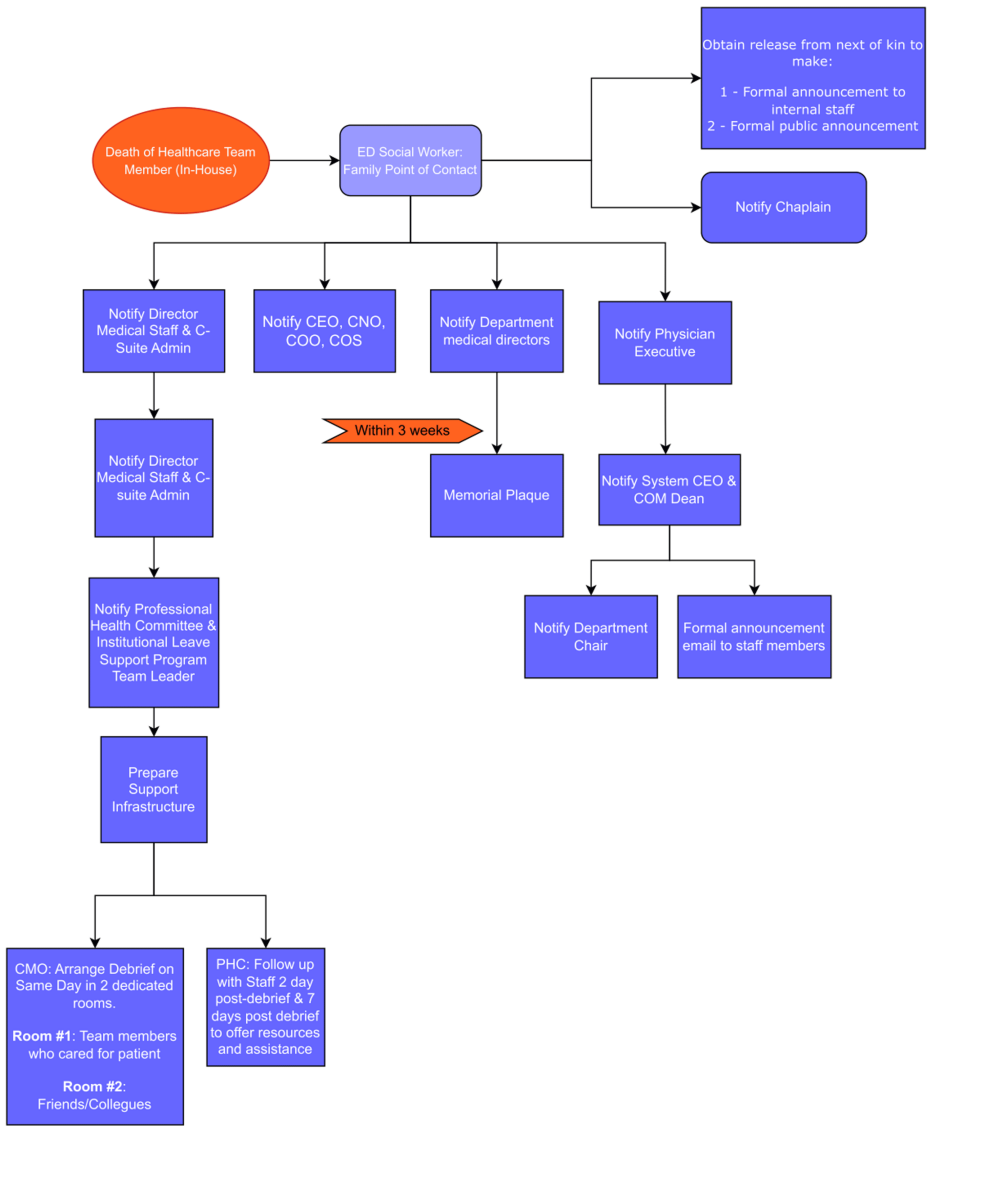
Organized diagram detailing plan for death of a healthcare team member within the hospital. T2M: Talk to Me, PHC: Professional Health Committee, CEO: chief executive officer, CMO: chief medical officer, CNO: chief nursing officer, COO: chief operating officer, COS: chief of staff.

**Figure 2:**
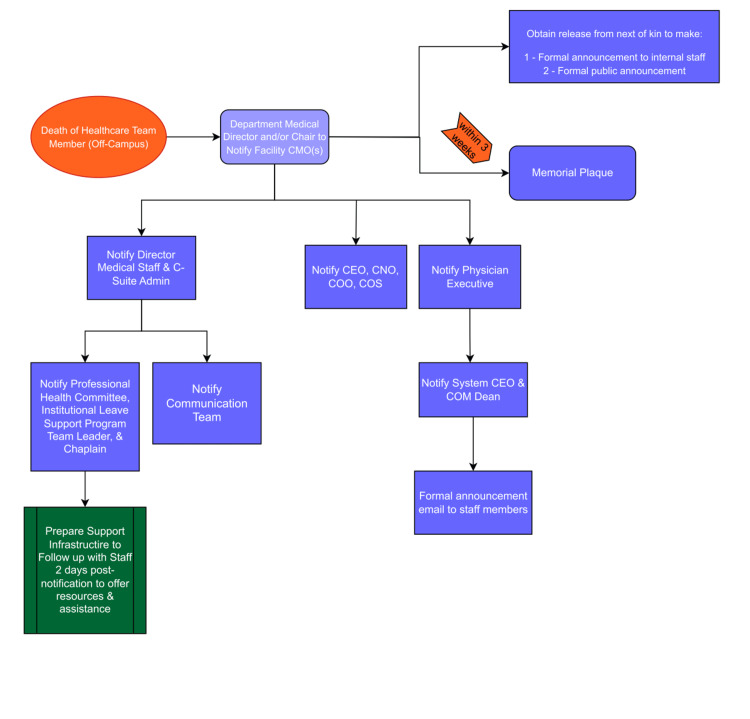
Organized diagram detailing plan for death of a healthcare team member that occurs off hospital campus. T2M: Talk to Me, PHC: Professional Health Committee, CEO: chief executive officer, CMO: chief medical officer, CNO: chief nursing officer, COO: chief operating officer, COS: chief of staff.

**Figure 3:**
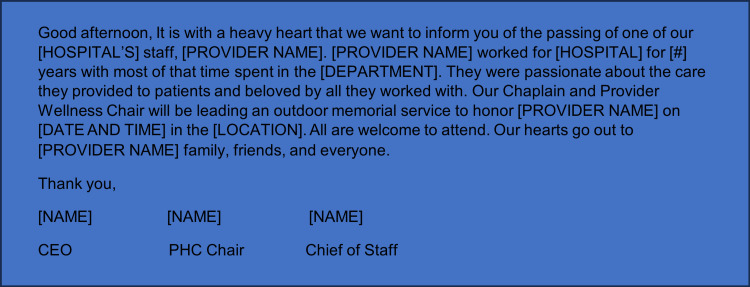
Example template email to inform hospital staff of team member death.

**Figure 4:**
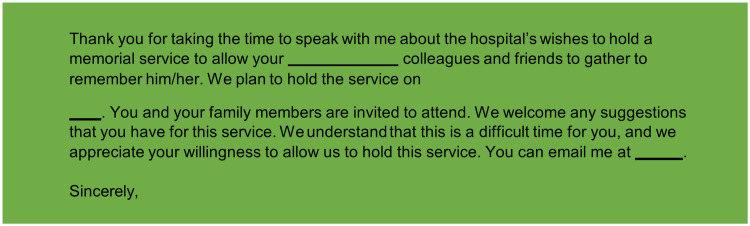
Template email for notification of memorial services held for team member’s death.

The resulting procedural framework includes two flow diagrams outlining response protocols based on the location of death, whether in the hospital or off-site (Figures 1, 2), a structured contact tree of key hospital leaders and support personnel involved in the response process (Table 1), template emails for initial death notification and memorial service communication (Figures 3, 4), and a directory of spiritual care contacts (Table 2) and staff mental health resources (Table 3). Stakeholder input was obtained from nursing leadership, hospital administration, medical staff services, chaplaincy, employee wellness, and frontline clinical providers.

## Results

The death of a healthcare team member is an event often surrounded by grief, stress, and uncertainty. In the absence of a structured plan, these circumstances may lead to confusion, miscommunication, and disrupted care. The implementation of a clear, organized framework can mitigate these issues and provide timely, compassionate support to affected individuals and departments. Our proposed procedural framework includes several key components. 

Flow diagrams for the notification process

We developed two flow diagrams detailing the stepwise notification and response process based on the location of the death. Figure 1 outlines the process for deaths occurring within the hospital, and Figure 2 outlines the process for deaths occurring off-site. Both diagrams ensure that key stakeholders are promptly notified and that appropriate actions are initiated.

Contact lists for notification and support

To facilitate efficient communication, we created a contact list including names, titles, and contact information for individuals and departments involved in the notification process (Table 1). Tables 2, 3 list spiritual care and mental health support resources available to staff.

**Table 1:** Organized contact list including position, name, email, and phone contact for organized notification system seen in Figure 1 and Figure 2. CEO: chief executive officer, CMO: chief medical officer, CNO: chief nursing officer, COO: chief operating officer, COS: chief of staff.

Position	Name	Email	Office Phone Number	Cell Phone Number
CEO	Name	email@domain.com	###-###-#####	###-###-#####
CNO	Name	email@domain.com	###-###-#####	###-###-#####
CMO #1	Name	email@domain.com	###-###-#####	###-###-#####
CMO #1	Name	email@domain.com	###-###-#####	###-###-#####
COO	Name	email@domain.com	###-###-#####	###-###-#####
COS	Name	email@domain.com	###-###-#####	###-###-#####
Physician Executive	Name	email@domain.com	###-###-#####	###-###-#####
COM Dean	Name	email@domain.com	###-###-#####	###-###-#####
Director of Medical Staff	Name	email@domain.com	###-###-#####	###-###-#####
C-Suite Admin	Name	email@domain.com	###-###-#####	###-###-#####
Professional Health Committee Co-Chair	Name	email@domain.com	###-###-#####	###-###-#####
Chaplain Director	Name	email@domain.com	###-###-#####	###-###-#####
Social Worker Director	Name	email@domain.com	###-###-#####	###-###-#####

**Table 2:** Organized contact list for hospital chaplains.

Name	Email	Office Phone Number	Cell Phone Number
Name	email@domain.com	###-###-#####	###-###-#####
Name	email@domain.com	###-###-#####	###-###-#####
Name	email@domain.com	###-###-#####	###-###-#####
Name	email@domain.com	###-###-#####	###-###-#####

**Table 3:** Organized list of support resources available to provide for hospital staff.

Resource	Description
Institutional leave support program	Continuous leave available for all team members in case of emotional distress caused by a work event. | Approved by director level or above.
National physician support line	A 24/7 support line for acute needs. | Website: https://physiciansupportline.com
Counseling and psychiatric services	Support available through Counseling and Psychiatric Services or Employee Assistance Counseling. | Resources for medical students, residents, fellows, and attending physicians are also available through Mental Health Services and fast-track appointments that can be scheduled by calling ###-###-#### or visiting the Hospital Outpatient Psychiatry website.
Hospital system mental health support	Resources include on-site, virtual, and one-on-one support. | Call 1-800-###-#### or visit the HospitalMentalHealthSupport.com website using username and password. | Video or text visits are also available at www.videotextvisits.com
Emergency/police	Dial 911 in case of emergencies.
Hospital security	Contact Hospital Security at ###-###-####.
24-hour crisis hotline(s)	County Crisis Response Line: ###-###-#### | National Crisis Response Line:1-800-274-TALK (8255) | Text Crisis line: 741-741
Graduate medical education (GME) mental health services	Resources and support available at mentalhealthservices.edu | GME Mental Health Services Director: [NAME]
Employee Insurance Provider Behavioral Health Plan	Contact Insurance Provider: #-###-###-####
Crisis center	Center for 24/7 drop-in psychiatric evaluations and crisis intervention services | Contact Crisis Response Center at ###-###-####.

Communication templates

To support timely and sensitive communication, we developed email templates for initial staff notification and memorial service announcements (Figures 3, 4).

Support resources for staff

Acknowledging the emotional toll on team members, we compiled a list of available support services, including mental health counseling, crisis lines, peer support, and spiritual care (Tables 2, 3).

These resources are designed not only to guide the institutional response but also to ensure continuity of care and support for the clinical team. For example, decisions regarding patient cancellations or provider coverage must be made quickly and with sensitivity. Our guidelines help streamline these logistical concerns while emphasizing privacy, compliance with institutional policy, and emotional support for grieving staff. These factors are especially important during a period often marked by intense distress and disorientation.

## Discussion

While being faced with the scenario of death among our own healthcare team members, we have come to appreciate the importance of understanding boundaries, personal differences, and the limitations of our system. We believe it is important to respect the individual who died, their family, and their colleagues, as they often have very personal relationships with the deceased. Some team members may need a leave of absence or may request to resign, in which case the issue regarding the continuation of patient care arises again and can further stress staffing and even increase costs to the hospital system. Providing appropriate resources is critical to support these team members. We observed that team members providing direct medical treatment of the individual who died may have a different experience than those who did not, underscoring the importance of providing clearly organized resources for support to all team members. While many healthcare workers have described how the COVID-19 pandemic has affected them, there are limited qualitative studies that highlight the response of nurses coping with grief [4]. Similarly, pediatric intensive care has also been shown to affect healthcare workers involved [5,6]. While primary action plans have been recommended, there are limited available examples for large hospital systems [7,8]. It is also critical to find ways to support leaders, as they are often the individuals running these protocols, and their grief may be delayed out of necessity of the job. This is consistent with findings on provider grief and the C.A.R.E. bereavement model [9,10]. Supporting leadership during bereavement has also been emphasized in the literature [11-15]. Our experience highlights the universal need for proactive planning and structured post-vention protocols across healthcare institutions.

## Conclusions

The circumstances surrounding the death of a team member may vary, but a well-developed, prepared, and organized approach to team member death may help avoid miscommunication and confusion and help provide services throughout the bereavement period. This structured approach can be used as a model for other healthcare facilities to create their own protocols. While grief is personal and complex, institutional responses play a vital role in promoting healing, resilience, and continuity of care. Future work may include formal evaluation of these protocols and adaptation to various healthcare settings.
